# Safety, tolerability, pharmacokinetics, and antiviral activity of the novel core protein allosteric modulator ZM-H1505R (Canocapavir) in chronic hepatitis B patients: a randomized multiple-dose escalation trial

**DOI:** 10.1186/s12916-023-02814-w

**Published:** 2023-03-16

**Authors:** Haiyan Jia, Jiajia Mai, Min Wu, Hong Chen, Xiaojiao Li, Cuiyun Li, Jingrui Liu, Chengjiao Liu, Yue Hu, Xiaoxue Zhu, Xiuhong Jiang, Bo Hua, Tian Xia, Gang Liu, Aiyun Deng, Bo Liang, Ruoling Guo, Hui Lu, Zhe Wang, Huanming Chen, Zhijun Zhang, Hong Zhang, Junqi Niu, Yanhua Ding

**Affiliations:** 1 Phase I Clinical Research Center, The First Hospital of Jilin University, No. 1 Xinmin Street, Changchun, Jilin Province China; 2 Gynecology and Obstetrics Center, the First Hospital of Jilin University, Changchun, China; 3 Shanghai Zhimeng Biopharma, Inc, 1976 Gaoke Middle Road, Suite A-302, Pudong District, Shanghai, China; 4 Department of Hepatology, Center of Infectious Disease and Pathogen Biology, The First Hospital of Jilin University, Changchun, China

**Keywords:** Antiviral therapy, Clinical trial, Hepatitis B, Pharmacokinetics

## Abstract

**Background:**

Hepatitis B virus (HBV) core protein-targeting antivirals (CpTAs) are promising therapeutic agents for treating chronic hepatitis B (CHB). In this study, the antiviral activity, pharmacokinetics (PK), and tolerability of ZM-H1505R (Canocapavir), a chemically unique HBV CpTA, were evaluated in patients with CHB.

**Methods:**

This study was a double-blind, randomized, placebo-controlled phase 1b trial in Chinese CHB patients. Noncirrhotic and treatment-naive CHB patients were divided into three cohorts (10 patients per cohort) and randomized within each cohort in a ratio of 4:1 to receive a single dose of 50, 100, or 200 mg of Canocapavir or placebo once a day for 28 consecutive days.

**Results:**

Canocapavir was well tolerated, with the majority of adverse reactions being grade I or II in severity. There were no serious adverse events, and no patients withdrew from the study. Corresponding to 50, 100, and 200 mg doses of Canocapavir, the mean plasma trough concentrations of the drug were 2.7-, 7.0-, and 14.6-fold of its protein-binding adjusted HBV DNA EC_50_ (135 ng/mL), respectively, with linear PK and a low-to-mild accumulation rate (1.26–1.99). After 28 days of treatment, the mean maximum HBV DNA declines from baseline were -1.54, -2.50, -2.75, and -0.47 log_10_ IU/mL for the 50, 100, and 200 mg of Canocapavir or placebo groups, respectively; and the mean maximum pregenomic RNA declines from baseline were -1.53, -2.35, -2.34, and -0.17 log_10_ copies/mL, respectively.

**Conclusions:**

Canocapavir treatment is tolerated with efficacious antiviral activity in CHB patients, supporting its further development in treating HBV infection.

**Trial registration:**

ClinicalTrials.gov, number NCT05470829).

**Supplementary Information:**

The online version contains supplementary material available at 10.1186/s12916-023-02814-w.

## Background

Chronic hepatitis caused by hepatitis B virus (HBV) infection is a major global health problem, with an estimated 0.6–1 million deaths per year [1, 2]. Emerging evidence supports the theory that persistent viral replication is associated with liver cirrhosis, liver cancer, and liver disease-related mortality [3, 4]. Although current antiviral therapies, such as monotherapy or combination therapy with pegylated interferon-alpha and nucleos(t)ide analogues, can effectively improve the quality of life of chronic hepatitis B (CHB) patients, they can only control HBV infection rather than curing infected hepatocytes due to the lack of a mechanism for complete HBV eradication. In addition, the emergence of drug resistance and viral relapse, as well as side effects such as thrombocytopenia and flu-like symptoms, pose major issues for these therapies [5].

Developing drugs targeting different HBV life cycle steps has become a critical approach for treating viral infections, such as CHB [6, 7]. There are numerous successful examples in this regard, such as the combination therapies developed for the treatments of human immunodeficiency virus and hepatitis C virus infection [8]. It is reasonable to expect that a similar strategy could be applied to the treatment of HBV infection. The HBV core protein (HBc) is a potential target for new anti-HBV therapy because it plays a critical role in various steps of the viral life cycle, including subcellular trafficking of the HBV genome, maintenance of the nuclear covalently-closed-circular DNA (cccDNA) pool, assembly of nucleocapsids, and epigenetic regulation of the viral and host genomes [9, 10]. HBV capsid assembly is a critical step in the propagation of the virus and is mediated by the core protein. Therefore, the development of pharmacological agents that target HBc, a structural component of the viral nucleocapsid, may be efficient for treating CHB caused by various HBV genotypes [10].

HBV core protein-targeting antivirals (CpTAs) are HBc-targeting small molecule compounds [9, 11–13]. CpTAs have two categories: (1) capsid assembly modulator-aberrant (CAM-A), which consists of heteroaryldihydro-pyrimidine derivatives and can misdirect HBc to form aberrant noncapsid polymers and lead to the degradation of the HBc [14, 15]; (2) CAM-empty (CAM-E), which consists of sulfamoylbenzamide derivatives and can accelerate the formation of morphologically normal HBV capsids that are nucleic acid-free [15, 16]. Both types of CpTAs (CAM-A and CAM-E) can disrupt HBV replication by preventing pregenomic RNA encapsidation during capsid assembly, leading to an increased yield of either aberrant or empty capsids, thereby affecting HBV replication and reducing pools of cccDNA during infection [17]. Currently, several CpTAs have been developed and studied in the early phases of clinical trials, such as GLS4, ABI-H0731, JNJ-6379, and RO7049389 [9, 11, 12].

Distinguished from the abovementioned CAM-A and CAM-E CpTAs, Canocapavir is a novel CAM-E CpTA with a pyrazole structure and a new binding site in the HBc [18]. Canocapavir also stimulates the intracellular accumulation of nonfunctional HBV capsids devoid of HBV DNA and pregenomic RNA (pgRNA). Canocapavir has shown potent and pan-genotypic anti-HBV effects in cell-based assays. Previously, in a phase 1a study, multiple doses (up to 300 mg) of Canocapavir (q.d. for 14 days) were found to be safe and well tolerated in healthy subjects. Its plasma exposure was well above its effective inhibitory concentration and increased in a dose-proportional manner [19] (See Additional file 1 for details). The present study aimed to evaluate the safety, tolerability, pharmacokinetics, and antiviral activity of Canocapavir in patients with CHB.

## Methods

### Study design

This was a randomized, open-label, placebo-controlled, phase 1b study (Clinical Trial Registration Number: NCT05470829; Chinese Clinical Trial Registration Number: CTR20210686), which was conducted at the Phase I Clinical Research Center, The First Hospital of Jilin University (Jilin, China), during the period from May to November of 2021. In order to minimize the safety risk of Canocapavir, an adaptive ascending dosing strategy was adopted for Canocapavir treatment in this study. The second and third assigned doses were initiated only after the safety data from the previous dose was reviewed and determined to be safe by the Principal Investigator and the Sponsor on day 8 postdosing (post drug administration days). Canocapavir was administered at the Clinical Research Center on days 1–8 and day 28 and at home on days 9–27 under the same conditions (fixed administration time and fasting condition). Patients were required to visit the facility for follow-up on days 15, 22, 27, 43 ± 1, and 57 ± 2 postdosing. The study protocol and informed consent forms were prepared according to the principles of the Declaration of Helsinki. The study protocol was approved by the Institutional Review Board of the First Hospital of Jilin University (approval number: 20Y201-006). All recruited patients provided written informed consent before participating in any study-related procedures.

### Participants

The main inclusion criteria of the participants were as follows: 1) adult subjects aged 18–65 years old who were treatment-naive or had terminated their treatment at least six months for nucleoside analog use or at least one year for interferon use prior to screening; 2) showing serum HBV DNA ≥ 2 × 10^4^ IU/mL for the subjects negative for hepatitis E surface antigen [HBeAg(-)] or serum HBV DNA ≥ 2 × 10^5^ IU/mL for the subjects with HBeAg( +); 3) level of alanine aminotransferase (ALT) less than 5 × the upper limit of normal. The main exclusion criteria were as follows: 1) with clinically significant acute or chronic liver diseases that are not caused by HBV infection, such as alcoholic liver disease, nonalcoholic fatty liver disease with severe or high severity, autoimmune liver disease, and Gilbert syndrome or other hereditary liver diseases; 2) cirrhosis and/or the value of liver stiffness measurement (LSM) ≥ 12.4 kPa (LSM ≥ 17.5 kPa when ALT > the upper limit of normal value). Additional inclusion and exclusion criteria are described in Additional file 1.

### Procedures

#### Randomization and blinding

**Fig. 1:**
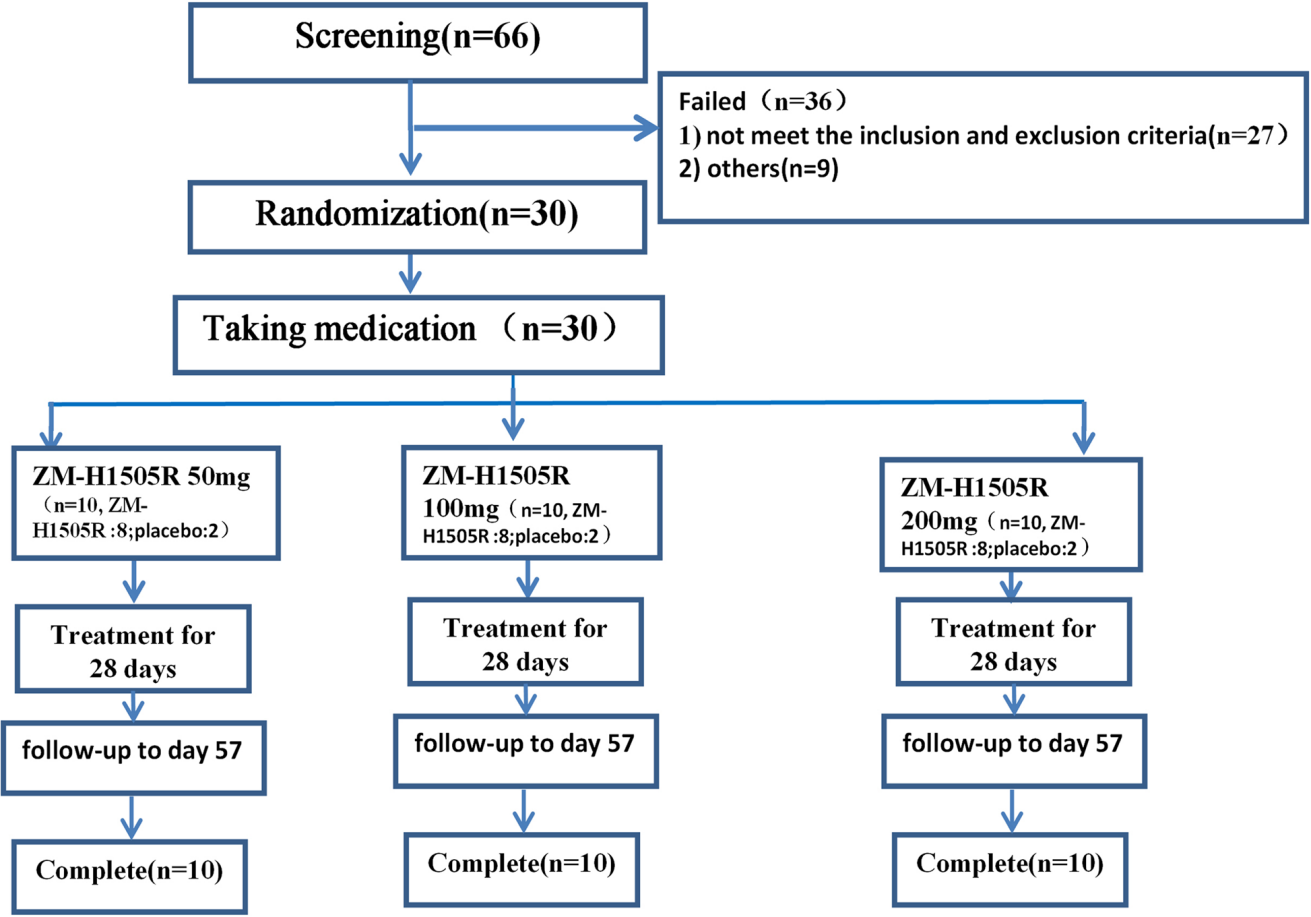
Design of this study

A total of 30 CHB patients were recruited in this study and were randomly assigned into three cohorts (*N* = 10, each). In these three cohorts, the patients (in a ratio of 4:1) received 50, 100, or 200 mg of Canocapavir or placebo once a day for 28 consecutive days under fasting conditions, followed by a 28-day follow-up (Fig. 1). The patients, care providers, and those assessing outcomes were blinded during the study. This random process was completed in an electronic data capture system, and the details are shown in Additional file 1.

#### Assessment of adverse reactions and drug tolerability

To examine possible adverse events (AEs) and adverse reactions (an adverse event is definitely, probably, or possibly related to the drug) in this study, the recruited patients underwent regular clinical laboratory testing, 12-lead electrocardiograms, examination of vital signs, abdominal ultrasound, and regular physical examinations, using the baseline data as a control. The severity of AEs and laboratory abnormalities were graded according to the Common Terminology Criteria for Adverse Events (CTCAE), version 5.0, which defines toxicity criteria. The AEs were coded using the Medical Dictionary for Regulatory Activities, version 24.1 (McLean, VA, USA).

### Pharmacokinetics

Blood samples for Canocapavir PK analysis were collected at different time points, and were collected and placed into a vacutainer with an anticoagulant (K_2_-EDTA), followed by centrifugation at 2,000 g and 4 °C for 10 min to collect the plasma.

### Antiviral activity

The changes in the serum levels of HBV DNA, HBV pgRNA, HBcrAg, HBsAg, HBsAb, HBeAg, HBeAb, and HBcAb at different time points were examined. Blood samples were collected on the initial screening day (baseline) and days 1, 8, 15, 22, 29, 43 ± 1, and 57 ± 2 postdosing. Blood samples for detecting viral resistance to Canocapavir were collected on day 1 (predosing), day 29 (24 h after day 28 dosing), and day 57 ± 2 postdosing.

### Statistical analysis

The following observed data were used for the analysis of the plasma PK parameters with the noncompartmental PK approach using WinNonlin, version 8.2, software (Pharsight Co., Mountain View, CA, USA): the maximum observed plasma concentration (Cmax), the time to maximum observed serum concentration (Tmax), area under the concentration–time curve from the time of dosing to the last time point with the measurable plasma concentration (AUC0-t) prior to the next dose, AUC from the time of dosing extrapolated to infinity (AUC_0-∞_), the terminal elimination half-life of the drug in plasma (t_1/2_), clearance (CL/F), volume (Vz/F), accumulation rate (RAC), and degree of fluctuation. All of these parameters in the treatment cohorts were analyzed using the descriptive statistics methods provided by SAS 9.1 software (Cary, NC, USA). The dose proportionality of Canocapavir was evaluated by the power model and the linear fixed-effect model. Descriptive analysis was used for safety, tolerability and antiviral activity indexes. Please see Additional file 1 for the detailed methodology.

## Results

### Demographics and disease characteristics of patients

This study was conducted during the period from May to November of 2021. Among the 66 patients that were screened, 30 fulfilled the inclusion and exclusion criteria, and no patient discontinued prematurely or was lost to follow-up during the study period. The recruited patients were all Chinese; 63.3% (19/30) of the patients were male, and 36.7% (11/30) of the patients were female. In general, the demographics and disease characteristics of the enrolled patients were matched among the different cohorts (Table 1). In total, 43.3% of the recruited patients were HBeAg(-). The mean baseline HBV DNA levels ranged between 6.04 and 7.16 log_10_ IU/mL, and the mean baseline ALT levels ranged between 30.89 and 46.65 U/L.

**Table 1:** Baseline demographic characteristics and clinical features

Baseline parameter (mean ± SD)	Canocapavir			Placebo *n* = 6
	50 mg *n* = 8	100 mg *n* = 8	200 mg *n* = 8	
Age, years	36.9 (9.08)	36.9 (6.15)	41.8 (10.91)	47.7 (10.86)
Sex (male/female)	6/2	3/5	6/2	4/2
Ethnicity (Han/other)	8/0	7/1	8/0	6/0
Weight (kg)	67.60 (10.645)	54.20 (7.648)	71.43 (6.443)	69.48 (7.51)
HBV DNA (log_10_ IU/mL)	6.68 (1.64)	7.16 (1.52)	6.04 (1.84)	6.42 (2.07)
HBV pgRNA (log_10_ copies/mL)	6.04 (1.66)	5.60 (1.68)	5.10 (2.00)	5.14 (2.79)
HBcrAg (log_10_ Ku/mL)	3.98 (2.16)	4.38 (1.86)	3.29 (2.04)	2.99 (2.45)
HBsAg (log_10_ IU/mL)	4.23 (0.62)	4.10 (1.00)	3.97 (0.75)	3.79 (1.17)
HBeAg (log_10_ PEI-U/mL)	3.06 (0.09)	3.09 (0.22)	2.05 (1.42)	2.77 (0.43)
HBeAg (positive/negative)	5/3	5/3	4/4	3/3
Fibroscan (kPa)	5.05 (0.42)	5.41 (2.18)	5.54 (1.10)	5.05 (1.01)
ALT (U/L)	30.89 (18.95)	46.65 (42.72)	41.13 (14.62)	35.98 (19.16)
AST (U/L)	27.09 (9.48)	52.61 (46.25)	33.06 (9.97)	30.75 (8.16)
genotype	B = 3; C = 5	B = 2; C = 6	B = 1; C = 7	B = 3; C = 3

*BMI* body mass index, *HBV* hepatitis B virus, *HBsAg* hepatitis B surface antigen, *ALT* alanine aminotransferase, *HBeAg* hepatitis B e antigen, *HBcrAg* hepatitis B core antigen, *pgRNA* pregenomic RNA

### Safety and tolerability

This study showed that Canocapavir was safe and tolerated over the 28 days of treatment. No serious AE occurred during the period of the trial. No patients withdrew from the study due to an AE, thus allowing the tolerability, antiviral, and PK analyses on data from all patients. A total of 76.7% (23/30) of the patients experienced 49 cases of AEs, in which 38 cases occurred in 75% of patients from the Canocapavir cohort, while 11 cases were from 83% of patients from the placebo cohort.

In total, 50% (15/30) of the subjects experienced 31 cases of adverse reactions, in which 24 cases occurred in 50% of patients from the Canocapavir cohort, while 7 cases were from 50% of patients from the placebo cohort. Most adverse reactions were mild or moderate in severity and resolved without treatment. The incidence rates of adverse reactions in each cohort were as follows: 50 mg, 37.5% (3/8); 100 mg, 62.5% (5/8); 200 mg, 50.0% (4/8); and placebo, 50.0% (3/6). The incidence of adverse reactions among patients who received Canocapavir was not significantly correlated to its increased dose (Table 2). In addition, the most common adverse reactions among patients who received Canocapavir were increased ALT (37.5%) and aspartate aminotransferase (AST) (25.0%). The most common adverse reactions among patients who received the placebo were also increased ALT (50.0%) and AST (50.0%). According to the criteria of CTCAE (version 5.0), two patients (6.7%) in this study had grade III adverse reactions, of which a decreased neutrophil count was observed in one subject receiving 50 mg of Canocapavir and hypertriglyceridemia was noted in one subject receiving 200 mg of Canocapavir. There were six grade II adverse reactions in three patients (10%), including a decreased white blood cell count (one case receiving 50 mg of Canocapavir and one case receiving 100 mg of Canocapavir), a decreased neutrophil count (one case receiving 100 mg of Canocapavir), a decreased ALT level (two cases in two patients receiving placebo), and an increased AST level (one case receiving placebo). There were 22 cases of grade I adverse reactions in 10 patients (33.3%) (Additional file 1: Table 1). Most AEs in this study spontaneously recovered or stabilized by the end of the study. The details are shown in Additional file 1.

**Table 2:** Frequency of adverse reactions in each treatment cohort [case, number of subjects (%)]

	Canocapavir						Placebo	
	50 mg, *n* = 8		100 mg, *n* = 8		200 mg, *n* = 8		*n* = 6	
	case	*n* (%)	case	*n* (%)	case	*n* (%)	case	*n* (%)
Total	6	3 (37.5)	10	5 (62.5)	8	4 (50)	7	3 (50)
Increased alanine aminotransferase	2	2 (25)	3	3 (37.5)	3	3 (37.5)	3	3 (50)
Increased aspartate aminotransferase	1	1 (12.5)	2	2 (25)	1	1 (12.5)	4	3 (50)
Decreased white blood cell count	1	1 (12.5)	1	1 (12.5)	0	0 (0)	0	0 (0)
Decreased neutrophil count	1	1 (12.5)	1	1 (12.5)	0	0 (0)	0	0 (0)
Increased γ-glutamyltransferase	0	0 (0)	1	1 (12.5)	0	0 (0)	0	0 (0)
Increased bilirubin	0	0 (0)	0	0 (0)	1	1 (12.5)	0	0 (0)
Increased creatinine	1	1 (12.5)	0	0 (0)	0	0 (0)	0	0 (0)
Decreased platelet count	0	0 (0)	1	1 (12.5)	0	0 (0)	0	0 (0)
Decreased serum phosphorus	0	0 (0)	0	0 (0)	1	1 (12.5)	0	0 (0)
Hypertriglyceridemia	0	0 (0)	0	0 (0)	2	1 (12.5)	0	0 (0)
Rash	0	0 (0)	1	1 (12.5)	0	0 (0)	0	0 (0)

### Pharmacokinetics

**Fig. 2:**
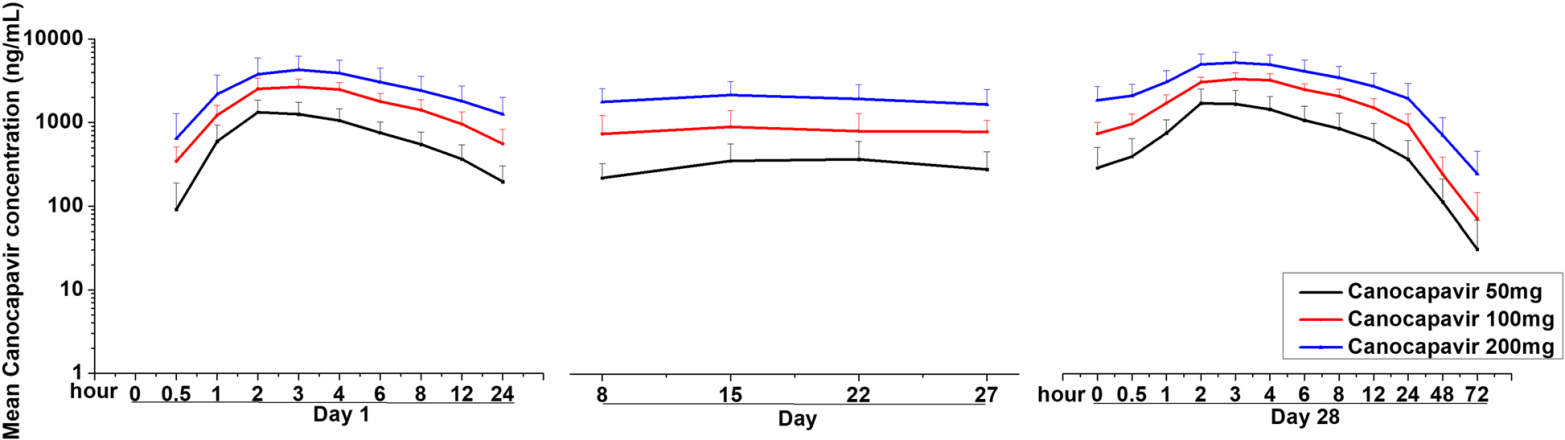
Mean values of the plasma concentrations of Canocapavir over time in each treatment cohort (mean ± standard deviation)

The concentration–time profiles of Canocapavir and the corresponding PK parameters in the patients who received different doses are shown in Fig. 2 and Table 3. Following the first and last dose of Canocapavir on days 1 and 28, the Canocapavir concentration increased after drug administration, reaching a maximum level at about 2–3 h (Fig. 2). A linear PK proportional relationship was observed between doses and plasma exposure of Canocapavir. The end-stage elimination of Canocapavir was a two-phase process with a mean t_1/2_ of 12.1–15.6 h after the last dosage. A steady state of QD dosing was achieved by day 8. The accumulation rate of the drug was small (1.26–1.99) in the patients who received different doses of Canocapavir, indicating that the drug accumulation rate was mild. On day 28, the mean plasma trough concentrations (C_trough_) in the patients who received 50, 100, or 200 mg of Canocapavir were 2.7-, 7.0-, and 14.6-fold of its protein-binding adjusted HBV DNA EC_50_ (135 ng/mL), respectively. The values of CLz/F and Vz/F were similar between the patients who received 50, 100, or 200 mg of Canocapavir. The degree of fluctuation was relatively small (121.8–203.5%).

**Table 3:** Pharmacokinetic parameters of Canocapavir on days 1 and 28 in each treatment cohort (mean ± SD)

	Day 1			Day 28		
	50 mg *n* = 8	100 mg *n* = 8	200 mg *n* = 8	50 mg *n* = 8	100 mg *n* = 8	200 mg *n* = 8
^a^T_max_ (h)	2.0 (2.0, 3.0)	3.0 (2.0, 4.0)	3.0 (2.0, 3.0)	2.5 (2.0, 3.0)	3.0 (2.0, 4.0)	3.0 (2.0, 4.0)
C_max_ (ng/mL)	1344.1 ± 498.2	2770.1 ± 652.5	4389.8 ± 1950.4	1752.3 ± 815.2	3398.7 ± 611.3	5330.6 ± 1656.4
t_1/2_ (h)^b^				13.0 ± 1.9	12.1 ± 3.7	15.6 ± 2.3
AUC_0-24 h_ (h·ng/mL)	11,732.9 ± 4933.2	28,447.1 ± 9350.9	50,875.4 ± 25,750.5	18,134.1 ± 9665.9	42,115.1 ± 9863.1	73,897.0 ± 29,108.4
AUC_0-t_ (h·ng/mL)	11,701.1 ± 4915.6	28,356.5 ± 9307.9	50,671.4 ± 25,634.8	24,716.5 ± 15,157.2	57,807.0 ± 16,630.8	114,031.6 ± 51,939.3
AUC_0-∞_ (h·ng/mL)^b^				25,625.6 ± 15,804.7	59,399.0 ± 18,159.0	120,090.0 ± 57,318.9
CL/F (L/h)^b^				3.4 ± 1.5	2.5 ± 0.6	3.3 ± 2.0
Vz/F (L)^b^				61.4 ± 23.0	42.3 ± 10.9	73.6 ± 43.4
C_trough_ (ng/mL)				289.8 ± 220.7	722.8 ± 256.6	1797.7 ± 933.9
degree of fluctuation (%)				203.5 ± 34.2	155.8 ± 21.9	121.8 ± 33.0
RAC_AUC0-24 h_				1.65 ± 0.85	1.56 ± 0.45	1.99 ± 1.81
RAC_Cmax_				1.44 ± 0.85	1.26 ± 0.29	1.52 ± 1.12

^a^Median (min–max);

^b^0–24 h blood samples were only collected on day 1. The values of CL/F, V_z_/F, t_1/2_, and AUC_0-∞_ were not well estimated due to the short elimination phase

Comparing the Canocapavir exposure (C_max_ and AUC) in the patients who received different doses of Canocapavir on days 1 and 28, the slope (90% confidence interval) in the power model was very close to 1 (regression coefficient: 0.82–1.14), suggesting linear PK of Canocapavir. The values of C_max_ and the AUC of Canocapavir were positively related to the drug doses (Additional file 1: Table 2).

### Antiviral activity

**Fig. 3:**
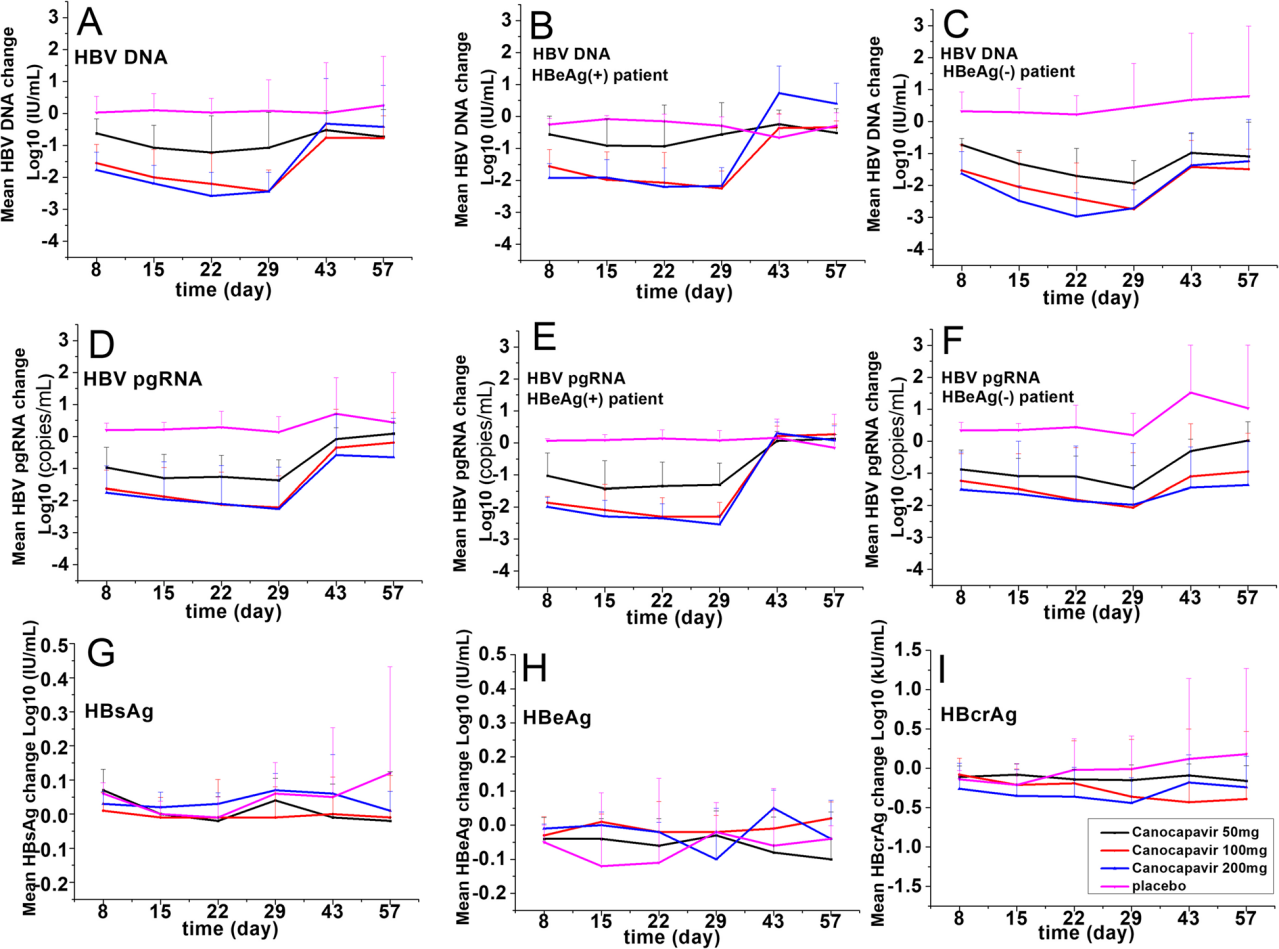
Changes of HBV virologic markers from baseline during treatment with Canocapavir or placebo. **A**–**C** Hepatitis B virus (HBV) DNA, **D**–**F** HBV pregenomic RNA (pgRNA), **G** hepatitis B surface antigen (HBsAg), **H** hepatitis e surface antigen (HBeAg), **I** hepatitis B core-related antigen (HBcrAg)

The level of HBV DNA decreased profoundly from baseline in the patients receiving three doses of Canocapavir but not in those receiving placebo. The HBV DNA level decreased greatly starting from day 8, with the average maximum decrease on day 22 or day 29. The HBV DNA level gradually returned to the baseline after drug withdrawal and recovered to the baseline at follow-up on day 57. Moreover, the HBV DNA level decreased overwhelmingly in patients who received 100 mg or 200 mg of Canocapavir compared to those who received 50 mg. The mean maximum HBV DNA decreases were -1.54, -2.50, -2.75, and -0.47 log_10_ IU/mL in the patients who received 50, 100, or 200 mg of Canocapavir or placebo, respectively. Regardless of the HBeAg status (either positive or negative), there was a declining trend in the HBV DNA level in the patients who received different doses of Canocapavir. However, among the patients who received the same dose of Canocapavir, the degree of HBV DNA decrease among the HBeAg( +) patients was slightly less than that of the HBeAg(-) patients. The numbers of patients (percentage) who received 50 mg, 100 mg, or 200 mg of Canocapavir or placebo and whose HBV DNA level was reduced below the lower limit of quantification after administration were 0, 1 (12.5%), 1 (12.5%), and 0, respectively. The reduction of HBV DNA below the lower limit of quantification only occurred in HBeAg(-) patients (Fig. 3 and Additional file 1: Fig. S1).

This study also showed that the HBV pgRNA level decreased profoundly from baseline in the patients who received different doses of Canocapavir but not in those who received the placebo. As mentioned above, the declining trend was similar to that of HBV DNA. The degree of HBV pgRNA decrease in the patients who received 100 mg or 200 mg of Canocapavir was similar, but it was deeper than that in the patients who received 50 mg. The mean maximum HBV pgRNA decreases were -1.53, -2.35, -2.34, and -0.17 log_10_ copies/mL in the patients who received 50, 100, or 200 mg of Canocapavir or placebo, respectively. The HBV pgRNA level decreased similarly in the patients with either HBeAg( +) or HBeAg(-) who received the same dose of Canocapavir. The numbers of patients (percentages) who received 50 mg, 100 mg, or 200 mg of Canocapavir or placebo and whose HBV pgRNA level was reduced below the lower limit of quantification (~ 2 log_10_ copies/mL) after administration were 1 (12.5%), 1 (12.5%), 4 (50.0%), and 0, respectively. A reduction of HBV pgRNA below the lower limit of quantification only occurred in HBeAg(-) patients (Fig. 3 and Additional file 1: Fig. S1). The rebound rate of the HBV pgRNA level was reversely dose-dependent, meaning that the higher dose was, the slower the rebound.

In addition, within the duration of treatment, the HBcrAg level in each dosage cohort decreased to a certain extent from baseline. The HBcrAg level decreased more in the patients who received 100 mg or 200 mg of Canocapavir than in those who received 50 mg of Canocapavir or the placebo. The maximum mean HBcrAg decreases were -0.30, -0.61, -0.51, and -0.38 log_10_ kU/mL in the patients who received 50 mg, 100 mg, or 200 mg of Canocapavir or placebo, respectively. Neither the HBsAg nor HBeAg levels decreased in the patients who received different doses of Canocapavir. However, they fluctuated around baseline (Fig. 3). There were no clinically significant changes in the levels of HBsAb, HBeAb, or HBcAb after administration of Canocapavir (data not shown). No resistance mutation that might be induced by Canocapavir treatment was detected in this study. One subject (E2003) was found to have a pre-existing substitution (P25S) in the HBc, which is known to reduce the susceptibility of the virus to CpTAs. After Canocapavir treatment, no decrease in HBV DNA and only a slight decrease in HBV pgRNA were observed in this patient. No new treatment-emergent resistance mutations were identified in this or other patients.

## Discussion

In this phase 1b study, we examined the safety, tolerability, PK profiles, and preliminary clinical efficacy of Canocapavir, a novel CAM-E CpTA for the treatment of patients with CHB. Our results showed that Canocapavir was safe and well tolerated in patients with CHB, and serum HBV DNA and HBV pgRNA were profoundly reduced after a 28-day treatment of Canocapavir.

Since CHB is a chronic disease and requires long-term treatment, safety is an important feature that a new drug must possess. In this study, an overall 50% incidence rate for adverse reactions was observed, which were not significantly correlated with the drug dosage. Most adverse reactions in this study were mild in severity, including elevation of ALT or AST. The data show that 12.5–37.5% of the patients who received Canocapavir had a grade I adverse reaction, while 50% of patients who received placebo had a grade I–II adverse reaction. These findings were similar with other CAM-E CpTAs, such as NVR 3–778 and ABI-H0731 [12, 20]. Therefore, even though the treatment duration in the current study is only about 4 weeks and treatment and observation of longer-term are definitely needed, Canocapavir seems safe and tolerated in CHB patients.

In our previous study [9], patients with high baseline ALT levels were prone to an ALT flare during antiviral treatment. This phenomenon was usually correlated with a decline in the HBV DNA and viral antigen levels, indicating that the occurrence of ALT flare is a therapeutic by-product [9]. It has also been reported that treatment with adefovir dipivoxil led to the decline of the HBV DNA level and a steady increase in the amount of T helper 1/2 cytokine-producing T cells and the levels of serum cytokines [21]. Nonetheless, an increased ALT level can be a double-edged sword, as it may reflect both immune system activation and liver cell damage (caused by viral infection or drug toxicity) [22]. We noticed that the ALT level of patient E3010, who received 100 mg of Canocapavir, was 144.9 U/L at baseline and increased gradually during the trial, reaching a maximum of 385.4 U/L on day 15, and the HBV markers in this patient decreased at the same time. The maximum decreases of HBV DNA, HBV pgRNA, HBcrAg, and HBsAg in this patient were -3.23 log_10_ IU/mL, -3.71 log_10_ copies/mL, -1.96 log_10_ kU/mL, and -0.22 log_10_ IU/mL, respectively. Because no abnormalities in bilirubin, albumin, or INR abnormality were found in this patient, the treatment-induced elevation of ALT may reflect a clearance of HBV-infected hepatocytes by an activated immune system [21, 22].

In contrast to this observation, we also noticed that in patient E4005, who received the placebo, the ALT level was 21.4U /L at baseline and increased gradually after day 31 (i.e., 3 days after the last dosing of the placebo), reaching to a maximum of 158.2 U/L on day 64. Similarly, in another patient (E4007) who also received the placebo, the ALT level was 65.8 U/L at baseline and increased gradually after day 15, reaching a maximum of 300.9 U/L on day 57 (Additional file 1: Fig. S2). The HBV DNA, HBV pgRNA, HBcrAg, and HBsAg levels in these two patients increased simultaneously. We interpret that the abnormally increased ALT in these two patients was caused by the aggravation of HBV infection, leading to liver damage [21, 22].

Chan et al. have reported that in cases of liver cirrhosis, the optimal cutoff values for the liver stiffness measurement were 8.4 kPa (98% sensitivity), 9.0 kPa (maximum sum of sensitivity and specificity), 13.4 kPa (94% specificity), and 13.4 kPa (maximum diagnostic accuracy, 85%) [23]. The mean liver stiffness measurement for all subjects in our study was 5.27 ± 1.30 kPa. Only one subject (E3007) in the Canocapavir (100 mg) group had a variation of liver stiffness measurement (10.5 kPa). The liver stiffness measurement values in the remaining subjects were less than 8.4 kPa. Moreover, one subject (E3007) had an abnormal ALT level (60 IU/mL) at the screening stage but did not have an AE during the clinical study. The mean liver stiffness measurement of these subjects was 5.06 ± 0.89 kPa, with a maximum liver stiffness measurement value (6.4 kPa). Therefore, this study could basically exclude the possibility that a higher frequency of ALT increases was caused by higher values for the liver stiffness measurement.

The occurrence frequency and rate of AEs, such as an increased ALT level, were similar among the different treatment groups. For example, the occurrence frequencies of an increased ALT level in the 50 mg, 100 mg, and 200 mg groups of Canocapavir and placebo were 2, 3, 3, and 3 cases, respectively (Table 2), indicating that the AE of an increased ALT was not related to the dose.

It has been reported that in the treatment with nucleos(t)ide analogues, a decrease of serum phosphorus levels and an increase of serum creatinine levels were observed; these can also happen in the presence of lactic acidosis and severe hepatomegaly with steatosis, including fatal cases. Severe acute exacerbations of hepatitis have been found in HBV-infected patients who have discontinued anti-hepatitis B therapy with nucleos(t)ide analogues [24]. In this study, abdominal ultrasound, regular physical examinations, and laboratory examinations were performed; therefore, liver function indexes (including bilirubin, AST, ALT, gamma-glutamyl transpeptidase, alkaline phosphatase, total protein, albumin, serum amylase, serum lipase, creatine kinase, and lactate dehydrogenase), coagulation function, kidney function (urea, creatinine), and ions (potassium, sodium, chlorine, calcium, and inorganic phosphorus) could be determined. We found an increase in grade I–II ALT and AST elevations; however, the rates were similar between the drug and placebo groups (Table 2). Increased levels of bilirubin, gamma-glutamyltransferase, and creatinine as well as a decreased level of serum phosphorus were found in the Canocapavir group, all of which were grade 1 without dose correlation and could be recovered automatically without treatment. Thus, in this study, there was no hepatic or renal toxicity similar to that observed with the nucleos(t)ide analogue treatment.

The PK profiles of Canocapavir showed rapid absorption (T_max_: 2–3 h) of this drug, with a slow plasma elimination (t_1/2_: 12.1–15.6 h). The accumulation of Canocapavir was mild, and its mean C_trough_ after dosing reached 2.7–14.6 times the EC_50_ (135 ng/mL ng/mL). Therefore, once-a-day administration is reasonable because of the large margin of the steady-state concentration of Canocapavir beyond its EC_50_ [8].

The conversion of HBV RNA to DNA occurs within viral core particles in the cytoplasm through reverse transcription, which is the prerequisite for both generations of progeny virus particles and replenishment of the HBV cccDNA pool in the nucleus. It has been postulated that complete and sustained disruption of the processes mentioned above may help accelerate the exhaustion of the cccDNA pool, eventually leading to permanent sterilization of HBV infection [25, 26]. Canocapavir promotes the production of the nonfunctional HBV capsid devoid of HBV DNA and pgRNA, thereby inhibiting the accumulation of cccDNA [18]. Although nucleoside analogues have little effect on reducing HBV RNA, the combinational use of a CpTA with a nucleoside analogue might have a more profound inhibitory effect on overall HBV replication than the single treatment with either of them. Therefore, it is reasonable to speculate that, given a long enough treatment, the combinational use of Canocapavir with a nucleoside analogue drug eventually will lead to a functional cure of CHB.

The changes in HBsAg, HBeAg, and HBcrAg levels have been studied for several CpTAs in phase I–II trials and generally are limited [11, 12, 20]. While we did not see significant changes in HBsAg and HBeAg after Canocapavir treatment in this study, we did observe some meaningful reduction in HBcrAg in some patients (see Fig. 3). The maximum mean HBcrAg decreases were -0.30, -0.61, -0.51, and -0.38 log_10_ kU/mL in patients who received 50 mg, 100 mg, or 200 mg of Canocapavir or placebo, respectively. Considering that some of HBcrAg (such as the core protein) are translated from pgRNA, disruption of the pgRNA package into a capsid (and its accumulation in the cytoplasm) by Canocapavir may interfere with the expression of these proteins. Studies are underway to characterize this mechanism.

One subject (E2003) was found to have a pre-existing substitution (P25S) in the HBc known to reduce the susceptibility of the virus to CpTAs [11], which may be responsible for the lower response of this patient to Canocapavir treatment. Indeed, after Canocapavir treatment, no decrease in HBV DNA and only a slight decrease in HBV pgRNA was observed in this patient. No new treatment-emergent resistance mutations were identified in this or other patients. The prevalence of the P25S resistance mutation was low (≤ 3.3%) in this study, suggesting a low risk of treatment failure due to pre-existing core inhibitor resistance [11, 12]. Nevertheless, identifying and excluding patients with pre-existing mutations known to cause reduced susceptibility to CpTAs may be needed in future studies of Canocapavir [9, 27].

This study provides the first clinical evidence that substantial inhibition of viral production in patients with CHB can be achieved by Canocapavir treatment. In a previous report, long-term entecavir therapy was associated with a decreased incidence of liver decompensation and hepatocellular carcinoma [28]. Since long-term oral antiviral therapy is associated with several disadvantages and virological relapse frequently occurs after termination of treatment, it is necessary to develop combinational therapies with HBV CpTA and other HBV-targeting drugs, which may be more efficient for the treatment of HBV patients [5, 25, 28].

### Study limitation

This study does have some limitations that must be addressed in future studies. These include a small number of patients, a short treatment and follow-up period, and the lack of analysis of immune-inflammatory factors. These limitations will be addressed in future multicenter studies of Canocapavir in more patients with a more extended treatment and follow-up period.

## Conclusions

Canocapavir was well tolerated. Most of the adverse reactions were grade I or II in severity. No dose-dependency was observed for either the frequency or intensity of AEs. Corresponding to 50–200-mg doses of Canocapavir, a linear PK profile and a low-to-mild accumulation rate (1.26–1.99) were notable. After oral dosing, significant margins were observed between plasma exposure of Canocapavir and its anti-HBV activity in vitro, supporting a once-a-day dosing in future trials. Canocapavir treatment for 28 days induced a substantial reduction of serum levels of both HBV DNA and HBV pgRNA in HBV patients. One subject (E2003) in the 50 mg of Canocapavir group did not respond to the treatment; this patient had a pre-existing mutation in the HBc (substitution of P25S), which is known to reduce the susceptibility of the virus to other CpTAs. The virus of the test subjects will need to be sequenced prior to treatment initiation, and monitored during and after treatment in future clinical studies. Taken together, these results support further clinical investigation of Canocapavir as a potent therapeutic agent for treating patients with CHB.

## Supplementary Information

**Additional file 1.**

## Data Availability

The data that support the findings of this study are available on request from the corresponding authors after the completion of the study. The data are not publicly available due to privacy or ethical restrictions.

## Abbreviations

HBV: Hepatitis B virus
CpTAs: Core protein-targeting antivirals
CHB: Chronic hepatitis B
PK: Pharmacokinetics
HBc: HBV core protein
cccDNA: Covalently-closed-circular DNA
CpTAs: HBV core protein-targeting antivirals
CAM-A: Capsid assembly modulator-aberrant
CAM-E: CAM-empty
pgRNA: Pregenomic RNA
LSM: Liver stiffness measurement
HBeAg: Hepatitis E surface antigen
ALT: Alanine aminotransferase
AEs: Adverse events
CTCAE: Common Terminology Criteria for Adverse Events
Cmax: The maximum observed plasma concentration
Tmax: The time to maximum observed serum concentration
AUC0-t: Area under the concentration–time curve from the time of dosing to the last time point with the measurable plasma concentration prior to the next dose
AUC0-∞: AUC from the time of dosing extrapolated to infinity
t1/2: The terminal elimination half-life of the drug in plasma
CL/F: Clearance
Vz/F: Volume
RAC: Accumulation rate
Ctrough: Trough concentrations
