# Supplementary material for: Safety, tolerability, pharmacokinetics, and antiviral activity of the novel core protein allosteric modulator ZM-H1505R (Canocapavir) in chronic hepatitis B patients: a randomized multiple-dose escalation trial
Source: BMC Med. 2023 Mar 16;21:98. doi: 10.1186/s12916-023-02814-w (PMC10022191; doi:10.1186/s12916-023-02814-w)
Supplement: Supplementary file 1 — Additional file 1. [file 12916_2023_2814_MOESM1_ESM.doc]

# Additional file 1

**Background**

To determine the anti-HBV activity of ZM-H1505R, the primary human hepatocyte (PHH) assay was performed *in vitro*. One day after infection with genotype D HBV, ZM-H1505R was added to the culture medium of PHH. The culture medium supplemented with ZM-H1505R was changed every two days. The culture supernatants were collected, of which the extracellular HBV DNA content was measured by qPCR. The results showed that the EC50 of ZM-H1505R was 12.32 nM and its EC90 value was 83.34 nM in regard to the inhibition of extracellular HBV DNA generation. The protein binding-adjusted HBV DNA EC50 was 135 ng/mL, and its EC90 was 937 ng/mL.

A phase Ia study to investigate the safety, tolerability, and pharmacokinetics (PK) of ZM-H1505R (Canocapavir) in healthy U.S. subjects has been reported (NCT04220801), which showed that the oral administration of single doses (25–450 mg) and multiple doses (75–300 mg) of Canocapavir were well tolerated. The most common AE found in that study was increased ALT. No dose dependency was observed in the incidence or intensity of AEs. The mean plasma AUC and C_max_ of Canocapavir increased in a dose-proportional manner. A significant margin (about 11.2–37.5 fold) was observed between the plasma exposure of Canocapavir and its anti-HBV activity EC50 *in vitro*. Food had an effect on the drug absorption. After multiple administration, the half-life of the drug was 12.2–21.7 h, and the accumulation rate was 1.7–2.3; therefore, QD administration was recommended for future studies ^[19]^.

The Canocapavir (75 mg) PK bridging study in healthy Chinese subjects was completed in China. The results were similar to those performed in the United States (the results have not yet been published). A phase Ib study of Canocapavir (50, 100, and 200 mg) in chronic hepatitis B patients in China was also conducted based on the PK, tolerance, and efficacy results in healthy subjects. Our study was a 28-day dosing phase Ib study in Chinese CHB patients.

Moreover, phase II clinical studies are ongoing, including a study to evaluate the efficacy and safety of ZM-H1505R in combination with ETV, as compared with ETV monotherapy in patients with CHB (NCT05484466).

**1. Methods**

The sentinel method was adapted for each dose cohort. First, two patients were enrolled to receive Canocapavir. The remaining 8 patients in each dose cohort were randomly assigned to Canocapavir or placebo at a ratio of 3:1. Eight random numbers were generated by stratified block randomization method using SAS 9.4 software. An independent statistician used SAS software to generate the random assignment table and imported the random assignment table into the electronic data capture system. The system assigned random numbers to the subjects based on their HBeAg positive/negative status and screening numbers.

Canocapavir and placebo were synthesized by Pharmaceutical Source Biotechnology (Qidong) Co. Ltd. and provided by Shanghai Zhimeng Biopharma. Inc., Shanghai, China (Batch No.: 5R90802-2, 5R90802-1, 5R90803-2, and 5R90803-1). The investigational drug was stored at room temperature and administered orally once daily in the morning under fasting conditions with 240 mL of water.

The concentration of Canocapavir was determined using a validated high-performance liquid chromatography-tandem mass spectrometry method of FRONTAGE Laboratories (Shanghai, China), with a detection range of 10–10,000 ng/mL.

Blood samples for Canocapavir PK analysis were collected at different time points, including preadministration (30 min predosing) and 0.5, 1, 2, 3, 4, 6, 8, 12, and 24 h postdosing on day 1; predosing on days 8, 15, 22, 27, and 28; and 0.5, 1, 2, 3, 4, 6, 8, 12, 24, 48, and 72 h postdosing on day 28.

The HBV DNA level was measured using a COBAS TaqMan kit (Roche Diagnostics, Pleasanton, CA, USA), according to the manufacturer’s instructions. The lowest detectable level using this kit was 20 IU/mL. The serum levels of HBeAg and HBsAg were quantitatively assessed by a chemiluminescence method using Abbott Architect assays (Abbott Diagnostics; Abbott Park, IL, USA). The lowest detectable level of the kit for HBeAg and HBsAg were 1 and 0.05 IU/mL, respectively. The viral resistance profile was analyzed by the polymerase chain reaction and DNA [sequencing](javascript:;). pgRNA was detected by quantitative polymerase chain reaction (AutoSAT, detection range:10^2^–10^8^ copies/mL), and HBcrAg was detected using a Lumipulse G HBcrAg assay (Fujirebio Europe, Belgium). All of the measurements mentioned above were performed either by Teddy Clinical Research Laboratory (Shanghai) or the Clinical Laboratory of the First Hospital of Jilin University.

**1.1 Additional inclusion criteria**

In addition to the main inclusion criteria mentioned in the manuscript, the following were additional inclusion criteria for the study participants: fully understanding the study contents, process, and potential adverse reactions; able to sign the informed consent forms prior to the study; able to complete the study in compliance with the protocol; agreed to adopt effective contraceptive measurements from screening to 6 months after the last administration (both the participant and their partners); weigh at least 50 kg for male subjects or 45 kg for female subjects; and have a body mass index (BMI) of 18–35 kg/m^2^, inclusive [BMI = weight (kg) / height^2^ (m^2^)]; nonpregnant and nonlactating females; with CHB showing hepatitis B surface antigen [HBsAg(+) and HBsAg/HBV DNA(+) for ≥6 months or IgM hepatitis B core; antibody (HBcAb)(-) but HBsAg(+)] detected in the screening;

treatment-naive or termination of treatment at least six months before screening for nucleoside analog use or one year for interferon use.

**1.2 Additional exclusion criteria**

In addition to the main exclusion criteria mentioned in the manuscript, the following were additional exclusion criteria for the study participants: major trauma or surgery within 3 months before screening; history of treatment that may interfere with drug absorption (e.g., subtotal gastrectomy); blood donation or massive blood loss (>450 mL) within 3 months prior to screening; any history of allergy suspected to be due to any component of the study drug or allergic constitution (allergic to multiple drugs and foods); history of narcotic drug intake or alcohol abuse; acute infection within 2 weeks before screening; suffer from serious diseases of the circulatory, respiratory, urinary, vascular, endocrine, immune, mental, or nervous system; history of myocardial infarction, unstable angina, percutaneous coronary intervention, coronary artery bypass grafting, heart failure of grade III or IV, or stroke within 6 months prior to screening; existing malignant tumors (except for skin nonmelanoma, cervical intraepithelial neoplasia, thyroid tumor, breast tumor, etc. after treatment with no signs of recurrence); any surgery or hospitalization anticipated during the study; history of treatment with immunosuppressants, immunomodulators (thymosin), or cytotoxic drugs within 6 months before study administration; history of treatment with strong CYP3A4 inhibitors or inducers within 2 weeks before screening; involved in any other drug administration study or clinical study of medical devices within 1 month before screening; involved in any clinical study of HBV virus nucleocapsid inhibitors in the past; any abnormality of electrocardiogram not suitable for the study as judged by a physician; for females, positive pregnancy test results or currently lactating; positive test results of HCV-Ab, HCV core antigen and HCV RNA (PCR), HIV antibody, or Treponema pallidum antibody further confirmed by a rapid plasma reagin test ( RPR test or RPR titer ) ; consumption of chocolate, any food or beverage containing caffeine or rich in xanthine, or any alcohol-containing products within 48 h before the initial administration; positive results of drug abuse tests (morphine, marijuana) or alcohol breath tests; other conditions in which the subjects are not suitable for the study in the opinion of investigators; clinically significant arrhythmia; history of risk factors for Torsade de Pointes syndrome; poorly controlled hypertension (systolic blood pressure ≥160 mmHg or diastolic blood pressure ≥100 mmHg), type 1 diabetes, or newly diagnosed or poorly controlled type 2 diabetes (HbA1c>8.5%) detected in the screening; and left ventricular ejection fraction <50%; co-infection with human immunodeficiency virus and/or hepatitis C virus; estimated glomerular filtration rate ˂60 mL/min/1.73 m^2^;

**Outcomes**

The co-primary endpoints were to evaluate the safety, tolerability, and PK parameters of multiple doses of Canocapavir in CHB patients. Secondary endpoints included evaluation of antiviral activity, including changes in the serum levels of HBV DNA, HBV pgRNA, hepatitis B core antigen (HBcrAg), HbsAg, hepatitis B surface antibody (HBsAb), HBeAg, hepatitis B e antibody (HBeAb), and HBcAb levels from baseline to the end of treatment and follow-up. Exploratory endpoints included detection of viral resistance developed during the treatment of Canocapavir.

**2. Results**

The changes in the laboratory testing items during treatment are shown in Additional file 1: Fig. S3, including the table listing the ALT, AST, γ-[glutamyltranspeptidase](javascript:;) (γ-GGT), alkaline phosphatase, and total bilirubin levels. The overall trend of the changes is best described as fluctuation. However, it should be noted that the serum levels of ALT, AST, and γ-GGT also increased on day 22 in the patients receiving the placebo.

The outcome of the AEs (increased ALT and AST) in one subject of the placebo cohort and the outcome of the AE (hyperglycemia) in one subject in the 200-mg Canocapavir cohort were “not recovered,” and the outcome of the AE (anemia) in one subject in the 50-mg Canocapavir cohort was “unknown” due to loss to follow-up. The other AEs in this study spontaneously recovered or stabilized by the end of the study. In these patients, six cases (six patients) of AEs required treatment with medication, including two patients (E2005 and E2006) who received 50 mg of Canocapavir were treated with dexamethasone rabies vaccine and immunoglobulin for urticaria and injury; one patient (E3004) who received 100 mg of Canocapavir was treated with levofloxacin for conjunctivitis; one patient (E3010) who received 100 mg of Canocapavir and two patients (E4005 and E4007) who received placebo were treated with glycyrrhizin tablets and silybin capsules (for protecting liver cells), and patient E4005 was also treated with entecavir for HBV infection.

All of the 30 enrolled patients were tested for the emergence of drug resistance mutations in the core gene, such as D29G, F23Y, I105F, L30F, P25A, P25G, T109I, T109M, T128I, T33N, T33Q, T33S, V124F, V124I, V124W, and Y118F. Only one patient (E2003, 3.3%) who received 50 mg of Canocapavir was found to have the P25S resistance mutation before (day 1) and after (days 29 and 33) administration of the test drug. This was an HBeAg (+) patient in which no decrease in the HBV DNA level was observed after administration, and HBV pgRNA was only slightly decreased on days 9, 22, and 43 (Additional file 1: Fig. S1).

**
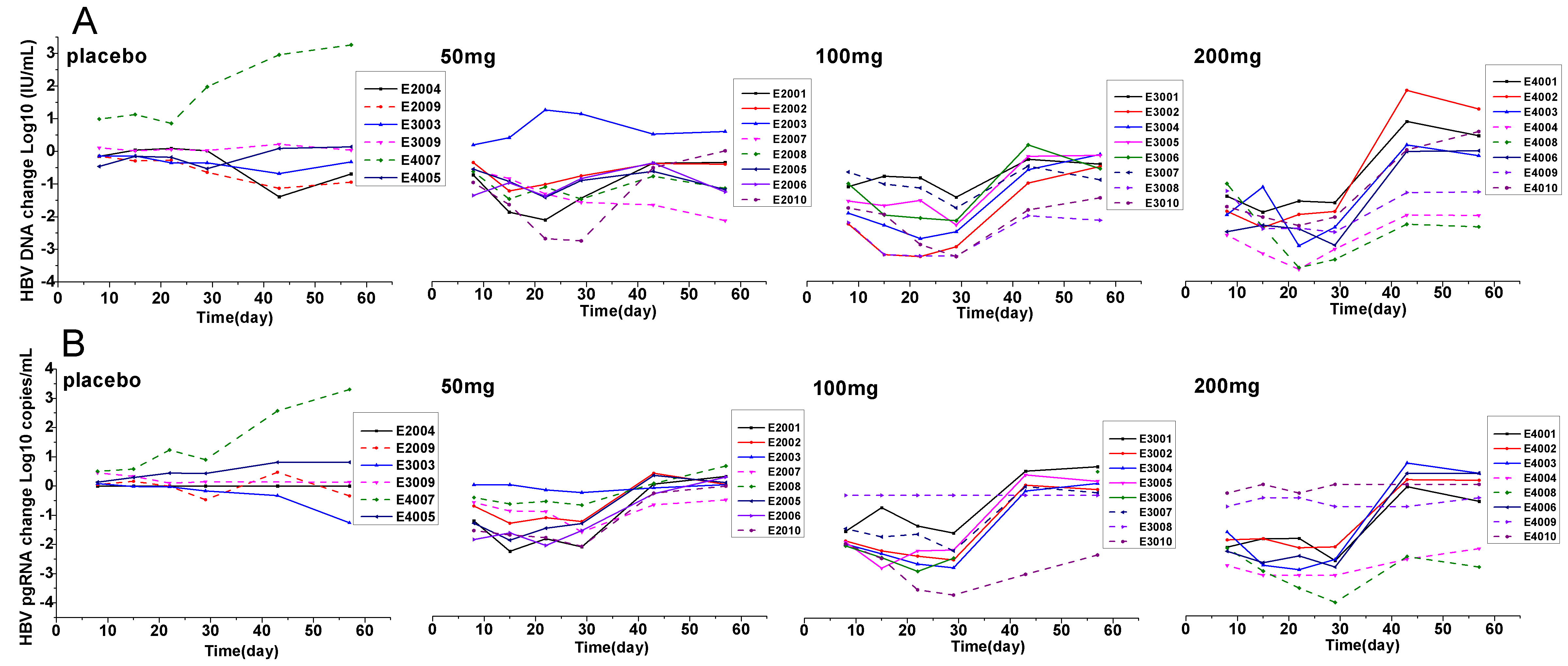
**

**Supplementary Figure 1.** Individual HBV DNA (A) and pgRNA (B) levels in individual HBeAg-positive (solid line) and HBeAg-negative (dotted line) patients during treatment with Canocapavir at doses of 0 mg (placebo), 50 mg, 100 mg, and 200 mg.


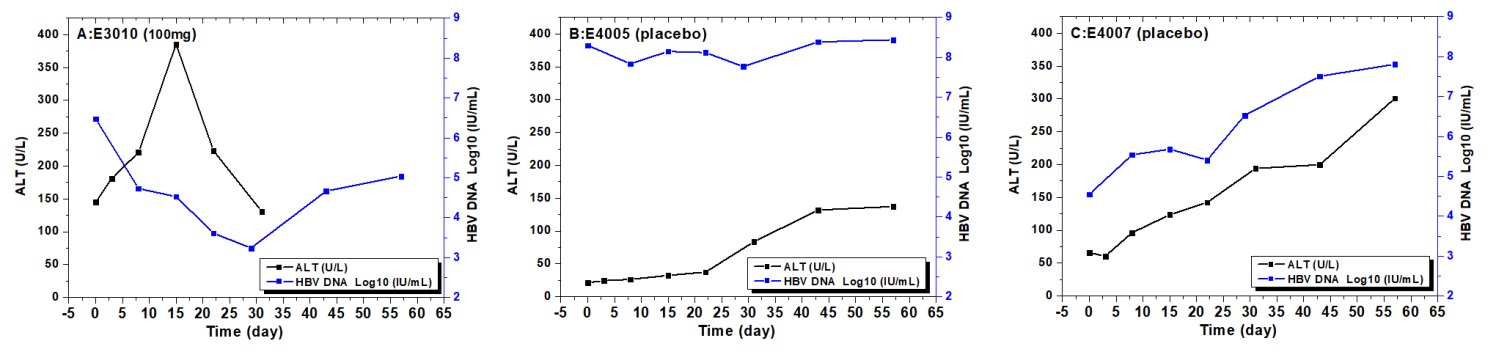


**Supplementary Figure 2.** Individual HBV DNA and ALT levels in individual patients during treatment with Canocapavir. (A) E3010, (B) E4005, and (C) E4007.


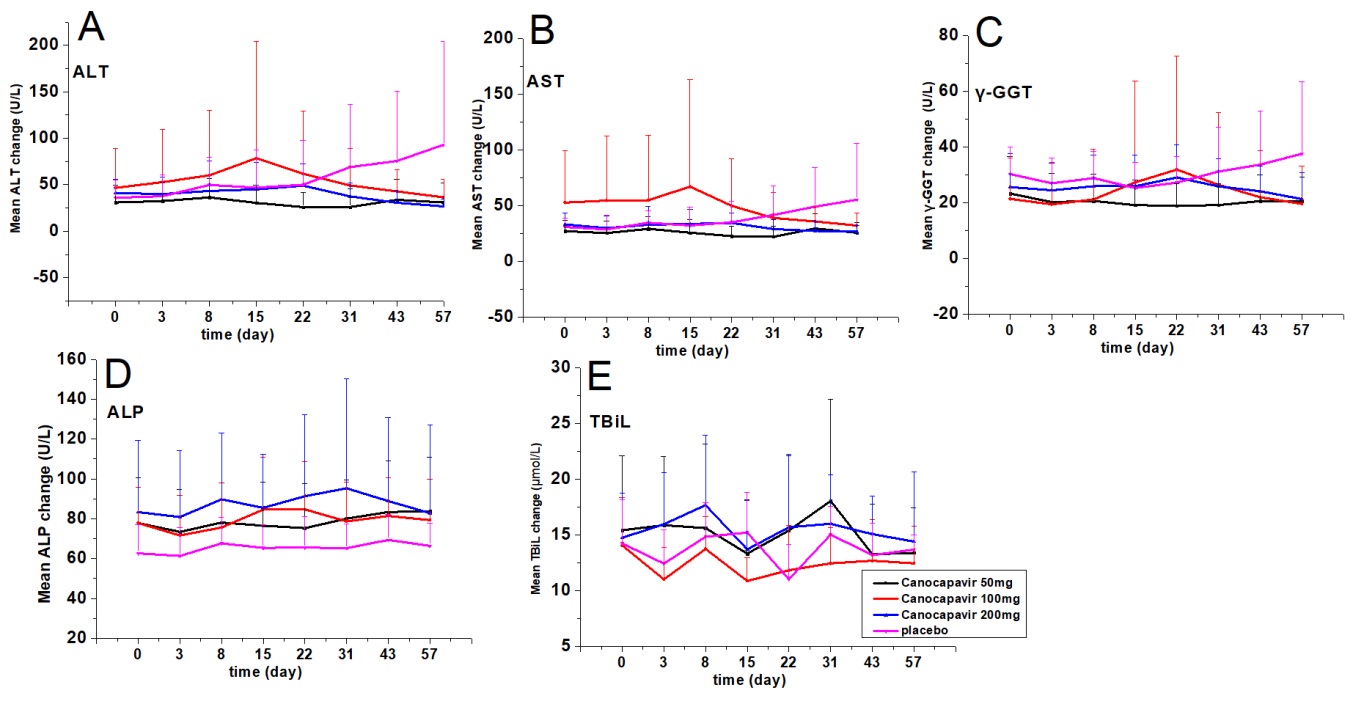


**Supplementary Figure 3.** Pattern changes in laboratory testing indices during treatment. (A) Alanine aminotransferase (ALT), (B) aspartate aminotransferase (AST), (C) γ-[glutamyltranspeptidase](javascript:;) (γ-GGT), (D) alkaline phosphatase (ALP), and (E) total bilirubin (TBIL).

Supplementary Table 1. The grade of adverse reactions in each treatment cohort [number of subjects (%)]

| Grade | Canocapavir | | | | | | Placebo | |
| --- | --- | --- | --- | --- | --- | --- | --- | --- |
|  | 50 mg, *n*=8 | | 100 mg, *n*=8 | | 200 mg, *n*=8 | | *n*=6 | |
|  | *n* | % | *n* | % | *n* | % | *n* | % |
| Total | 3 | 37.50% | 5 | 62.50% | 4 | 50.00% | 3 | 50.00% |
| I | 2 | 25.00% | 4 | 50.00% | 3 | 37.50% | 1 | 16.70% |
| II | 0 | 0 | 1 | 12.50% | 0 | 0 | 2 | 33.30% |
| III | 1 | 12.50% | 0 | 0 | 1 | 12.50% | 0 | 0 |
| Increased alanine aminotransferase |  |  |  |  |  |  |  |  |
| I | 2 | 25.00% | 3 | 37.50% | 3 | 37.50% | 1 | 16.70% |
| II | 0 | 0 | 0 | 0 | 0 | 0 | 2 | 33.30% |
| Increased aspartate aminotransferase |  |  |  |  |  |  |  |  |
| I | 1 | 12.50% | 2 | 25.00% | 1 | 12.50% | 2 | 33.30% |
| II | 0 | 0 | 0 | 0 | 0 | 0 | 1 | 16.70% |
| Decreased white blood cell count |  |  |  |  |  |  |  |  |
| II | 1 | 12.50% | 1 | 12.50% | 0 | 0 | 0 | 0 |
| Decreased neutrophil count |  |  |  |  |  |  |  |  |
| II | 0 | 0 | 1 | 12.50% | 0 | 0 | 0 | 0 |
| III | 1 | 12.50% | 0 | 0 | 0 | 0 | 0 | 0 |
| Increased γ-glutamyltransferase |  |  |  |  |  |  |  |  |
| I | 0 | 0 | 1 | 12.50% | 0 | 0 | 0 | 0 |
| Increased bilirubin |  |  |  |  |  |  |  |  |
| I | 0 | 0 | 0 | 0 | 1 | 12.50% | 0 | 0 |
| Increased creatinine |  |  |  |  |  |  |  |  |
| I | 1 | 12.50% | 0 | 0 | 0 | 0 | 0 | 0 |
| Decreased platelet count |  |  |  |  |  |  |  |  |
| I | 0 | 0 | 1 | 12.50% | 0 | 0 | 0 | 0 |
| Decreased serum phosphorus |  |  |  |  |  |  |  |  |
| I | 0 | 0 | 0 | 0 | 1 | 12.50% | 0 | 0 |
| Hypertriglyceridemia |  |  |  |  |  |  |  |  |
| III | 0 | 0 | 0 | 0 | 1 | 12.50% | 0 | 0 |
| Rash |  |  |  |  |  |  |  |  |
| I | 0 | 0 | 1 | 12.50% | 0 | 0 | 0 | 0 |

Supplementary Table 2. Linear regression analysis of Canocapavir exposure on days 1 and 28 (power model).

| Time | PK parameter | [regression](javascript:;) [coefficient](javascript:;) | 90% [confidence interval](javascript:;) |
| --- | --- | --- | --- |
| Day 1 | C_max_ | 0.82 | 0.53–1.10 |
|  | AUC_0-t_ | 0.99 | 0.65–1.32 |
| Day 28 | C_max_ | 0.83 | 0.60–1.05 |
|  | AUC_0-t_ | 1.14 | 0.83–1.43 |
|  | AUC_0-∞_ | 1.14 | 0.83–1.45 |
